# Recent Advances in Plant Early Signaling in Response to Herbivory

**DOI:** 10.3390/ijms12063723

**Published:** 2011-06-07

**Authors:** Gen-Ichiro Arimura, Rika Ozawa, Massimo E. Maffei

**Affiliations:** 1 Global COE Program: Evolution and Biodiversity, Graduate School of Science, Kyoto University, Kyoto 606-8502, Japan; 2 Center for Ecological Research, Kyoto University, Otsu 520-2113, Japan; E-Mail: ozawar@ecology.kyoto-u.ac.jp; 3 Plant Physiology Unit, Department of Plant Biology and Innovation Centre, University of Turin, 10135 Turin, Italy; E-Mail: massimo.maffei@unito.it

**Keywords:** effector, elicitor, herbivore, plant defense response, protein kinase, volatile organic compound (VOC)

## Abstract

Plants are frequently attacked by herbivores and pathogens and therefore have acquired constitutive and induced defenses during the course of their evolution. Here we review recent progress in the study of the early signal transduction pathways in host plants in response to herbivory. The sophisticated signaling network for plant defense responses is elicited and driven by both herbivore-induced factors (e.g., elicitors, effectors, and wounding) and plant signaling (e.g., phytohormone and plant volatiles) in response to arthropod factors. We describe significant findings, illuminating the scenario by providing broad insights into plant signaling involved in several arthropod-host interactions.

## 1. Recognition System of Arthropod Herbivores in Plants

### 1.1. Herbivore-Derived Elicitors

Coordination of defensive actions against attacking pests results from interactions between the plant and herbivore-derived elicitors and effectors which are followed by rapid activation of sophisticated plant signaling cascades. However, the molecular mechanisms in the hosts that regulate the balance between activation and suppression of resistance are not fully understood. Despite the high number of known plant responses to herbivory, there are only a few known classes of animal-derived defense elicitors [1].

The first fully characterized herbivore-derived elicitor was volicitin [*N*-(17-hydroxylinolenoyl)-Lglutamine], a hydroxy fatty acid-amino acid conjugate (FAC), which was identified in beet armyworm (*Spodoptera exigua*) oral secretions [2]. The biological functions of FACs on plants and FACs variation patterns in lepidopteran species have been intensively studied and recently reviewed [3]. For example, it was found that FACs introduced into wounds during feeding are rapidly metabolized by lipoxygenases in the octadecanoid pathway to form additional active elicitors [4–6]. Plant cell plasma transmembrane potential (Vm) depolarization may be triggered by FAC-type elicitors due to their amphiphilic nature and, thus, detergent-like potential ion fluxes induced by oral secretions initiate Vm depolarization and, as a consequence, the opening of voltage-dependent Ca^2+^ channels to transmit the signal [7]. Until now, massive investigations have been carried out with oral secretions of herbivores, and some succeeded in identifying other elicitors and other herbivore-associated molecules, such as caeliferins [8], β-glucosidase from cabbage white butterfly (*Pieris brassicae*) [9], benzyl cyanides from *P. brassicae* [10], disulfooxy fatty acids (caeliferins) from the American bird grasshopper (*Schistocerca americana*) [8] and inceptins from fall armyworm (*Spodoptera frugiperda*) [11], and also the effect of microbes on the plant surface that may alter plant defensive pathways have recently been reported (reviewed in [12]). Inceptin [^+^ICDINGVCVDA^−^] and the related peptides [^+^(GE)ICDINGVCVDA^−^] are derived from chloroplastic ATP synthase gamma-subunit regulatory regions. These peptides elicit rapid and sequential production of phytohormones, and consequently volatile emissions [13]. In contrast to caterpillars, however, little is known about oral elicitors from sucking arthropods (spider mites and aphids). It has very recently been proposed that the release of aphid elicitors (e.g., oligogalacturonides) due to cell wall digestion by gel saliva enzymes may induce Ca^2+^ influx [14].

In addition, egg deposition might also elicit plant responses [15]. Induction of plant defensive responses by insect egg deposition is caused by the egg or egg-associated components of several insects, although the responsible chemistry has been identified only in bruchid beetles: long-chain α, γ-monounsaturated C_22_ diols and α, γ-mono- and di-unsaturated C_24_ diols, mono- or diesterified with 3-hydroxypropanoic acid [16]. Similarly, it is possible that there are potent elicitors released by herbivorous arthropods during tarsal contact with a plant but they have not so far been found [15].

### 1.2. Suppression of Plant Defenses by Herbivores

**Figure 1:**
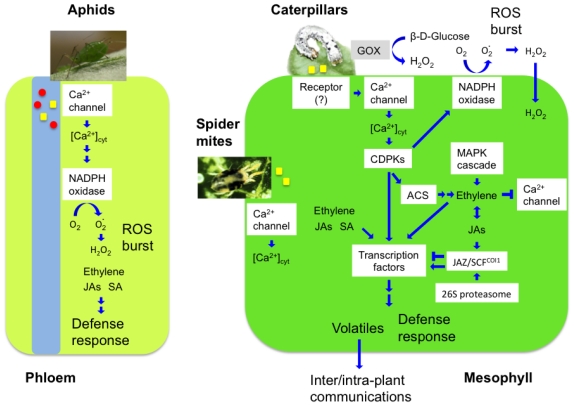
Model of the signaling network for plant defense responses to chewing arthropod (caterpillars) and sucking arthropods (aphids and spider mites). Arrows and bars indicate positive and negative interactions, respectively. The overall scenario may differ in certain plant taxa. However, in general, chewing arthropods induce JA-dependent defense responses, whereas piercing-sucking arthropods frequently induce SA-dependent defense responses. Red circles and yellow square molecules indicate oral factors of arthropods (effectors and elicitors, respectively). Abbreviations: ACS, 1-aminocyclopropane-1-carboxylate (ACC) synthase; CDPKs, Ca^2+^-dependent protein kinases; GOX, glucose oxidase; JAs, jasmonates; MAPK, mitogen-activated protein kinase; ROS, reactive oxygen species; SA, salicylic acid.

Although some pathogens suppress these defenses by interfering with signaling pathways involved in the defense, evidence of such interference is scarce for herbivores. However, feeding by herbivorous arthropods, whether defoliation or by feeding on specific tissues (e.g., phloem or xylem), triggers a complex and interacting array of molecular and physiological responses in plants. These responses potentially reduce host resistance and even photosynthesis [17]. Suppression of host defenses and alteration of host plant phenotypes occur widely in a large array of plant-pest (especially, plant-pathogen) interactions and involve secretion of molecules (effectors) that modulate host cell processes [18,19]. Massive proteomic and transcriptomic studies were carried out with lepidopteran salivary glands, and some succeeded in identifying key components of saliva. Mandibular glands of *Helicoverpa zea* were found to secrete salivary glucose oxidase (GOX) [20], an enzyme which functions as an effector that suppresses the induced defenses of the host plant by contributing to the initial oxidative burst of H_2_O_2_ observed in leaves damaged by herbivores [21,22] (Figure 1). Eichenseer *et al.* found a significant relationship between host range breadth and GOX activities, where highly polyphagous species show relatively high levels of GOX compared to species with more limited host range [22].

Recently, it has been demonstrated that egg-derived elicitors trigger the suppression of defenses against chewing herbivores in Arabidopsis. This process is mediated by salicylic acid (SA), as evidenced by the lack of gene suppression and the absence of enhanced susceptibility in *sid2-1* mutants [23]. Herbivore species that belong to different feeding guilds, such as parenchymal cell content feeders and phloem feeders, may trigger different plant responses. In Arabidopsis plants infested by the phloem-feeding silverleaf whitefly (*Bemisia tabaci*) SA-responsive gene transcripts accumulated locally and systemically, whereas jasmonic acid (JA)- and ethylene-dependent RNAs were repressed or not modulated [24]. Furthermore, *B. tabaci* was found to interfere with the indirect defense of Lima bean plants in response to generalist spider mites (*Tetranychus urticae*) through inhibition of the JA signaling pathway induced by the latter [25]. *Tetranychus evansi* suppresses the induction of the SA and JA signaling routes involved in induced plant defenses in tomato [26]. Moreover, distinct variations within a single herbivore species, the spider mite *T. urticae*, in traits that lead to resistance or susceptibility to JA-dependent defenses of a host plant and also in traits responsible for induction or repression of JA defenses have been demonstrated [27]. Aphids, similarly to plant pathogens, deliver effectors inside their hosts to manipulate host cell process enabling successful infestation of plants [28]. Plant disease resistance (R) proteins that recognize plant pathogens and those that confer resistance to aphids share a similar structure, and contain a nucleotide binding site (NBS) domain and leucine rich repeat (LRR) regions [29,30]. Recently, a functional genomics approach for the identification of candidate aphid effector proteins from the aphid species *Myzus persicae* (green peach aphid) based on common features of plant pathogen effectors has been developed [28]. Data mining of salivary gland expressed sequence tags (ESTs) made it possible to identify 46 putative secreted proteins from *M. persicae*. Functional analyses of these proteins showed that, among them, Mp10 induced chlorosis and weakly induced cell death in *Nicotiana benthamiana*, and suppressed the oxidative burst induced by the bacterial PAMP flagellin 22 (flg22). In addition, using a medium throughput assay based on transient overexpression in *N. benthamiana*, two candidate effectors (Mp10 and Mp42) have been identified as reducing aphid performance, whereas MpC002 enhanced aphid performance [28]. Overall, aphid-secreted salivary proteins share features with plant pathogen effectors and therefore may function as aphid effectors by perturbing host cellular processes.

Many other suppressing systems have been described. The larvae of several lepidopteran species including *Pieris rapae* and *P. brassicae* contain a nitrile-specifier gut protein that detoxifies the breakdown products of glucosinolates, which are the major insect deterrents in Arabidopsis [31]. The cytochrome P450 monooxygenase gene superfamily in *Papilio* butterflies is used against furanocoumarins [32] and the flavin-dependent monooxygenase system of the arctiid moth *Tyria jacobaeae* is used against pyrrolizidine alkaloids [33]. Nematode effectors play roles in causing plant susceptibility. A direct interaction was found between a nematode-secreted peptide and a plant-regulatory protein [34].

### 1.3. Plant Damaged-Self Recognition

The ability to distinguish between self and non-self is highly conserved in living organisms, including plants. Plants respond differently to self- and non-self signals and they may also be able to respond differentially based on levels of relatedness [35]. Research on the general processes during resistance induction has recently been re-directed towards elicitors that stem from the damaged plant itself. The first level of the plant immune system provides recognition of a broad spectrum of microorganisms, whereas the second level allows certain plants to detect specific pathogen strains—a phenomenon also referred to as “gene-for-gene resistance” [36]. Recently, Heil formulated the concept of “plant damaged-self recognition” which is based on the observation that animal feeding on plant tissues generates the disruption and disintegration of plant cells [1]. This damage moves plant molecules outside the protoplast and releases cell fragments that become exposed to enzymes that, in the intact cell, are localized to different compartments. These released molecules are signatures of “damaged self” and may include elicitors of plant defense responses [1]. Thus, whereas the herbivore has developed methods of feeding, the plant has evolved mechanisms for perception of attack and activation of defense responses, based on surveillance of its own tissue. However, the wounding alone can induce self-recognition molecules. Upon wounding of tomato plants, the plant peptide signal systemin is released from its precursor and, through receptor-mediated events, initiates the JA signaling, producing protease inhibitors and other defense compounds that protect the plant from further attack [37]. Recently, a peptide which is processed from a unique region of an extracellular subtilisin-like protease (subtilase) has provided insight into the mechanism by which host plant-derived, damage-associated signals mediate immune responses [38]. It has also been demonstrated that plants respond differently to volatile cues from self and non-self ramets that have been experimentally clipped [39]. Thus, the ability to recognize self-produced plant molecules elicited by or released from the plant cell as a consequence of herbivory as well as the ability of kin selection, and self, non-self discrimination is opening interesting new horizons in the study of plant interactions with the surrounding biotic and abiotic environment.

## 2. Protein Phosphorylation Signaling: MAPK *vs.* CDPK

Internal signaling requires that the signal transduction pathways recognize signaling molecules (*i.e*., elicitors) such as those described above and transfer the signal to the nuclear genomic machinery through a comprehensive network of interacting pathways downstream of the sensors/receptors. A large array of interconnected signaling pathways, including protein kinase cascades and their downstream responses, evoke secondary feedback signaling to regulate the metabolic balance during the defense response.

At least the mitogen-activated protein kinases (MAPKs) and Ca^2+^-binding sensory proteins concomitantly and independently play important roles in mediating herbivory responses. Herbivory- or wounding-related MAPKs, SA-induced protein kinase (SIPK) and wound-induced protein kinase (WIPK), were the first MAPKs identified in tobacco [40]. Transcripts of the WIPK gene begin to accumulate one minute after mechanical wounding, leading to the induced production of JA-inducible gene transcripts [40,41]. In addition, SIPK is also known to be involved in both JA and ethylene production [42,43], whereas activation of SIPK after wounding is associated with increased tyrosine phosphorylation but not with increases in SIPK mRNA or protein levels [44]. Kandoth *et al*. reported that co-silencing of *MPK1* and *MPK2* (orthologues of *WIPK* and *SIPK* genes) in tomato overexpressing prosystemin weakens proteinase inhibitor-associated defense against the specialist herbivore *Manduca sexta* [45].

In addition, plants possess several classes of Ca^2+^-binding sensory proteins, including calmodulins (CaMs), calmodulin-like proteins, calcineurin B-like proteins, and Ca^2+^-dependent protein kinases (CDPKs) [46]. Following insect attack, Arabidopsis CPK3 and CPK13 play a role in the transcriptional activation of plant defensin gene *PDF1.2* [47,48]. This cascade is not involved in the phytohormone (JA and ethylene)-related signaling pathways, but rather directly impacts transcription factors for defense responses. In turn, those CDPKs are directly involved in transcriptional activation of *PDF1.2* by phosphorylating a heat shock transcriptional factor (HsfB2a) in herbivore-infested plants. These findings are in line with those about tobacco CaM (NtCaM13) which has an independent action from the JA and ethylene signaling pathways for basal defense against necrotrophic pathogens [49]. In contrast to those examples, tobacco CDPK (NtCDPK2) was suggested to participate in the synthesis of ethylene and oxylipins (JA and its related compounds) and, moreover, in cross-talk with the WIPK/SIPK cascade activated by pathogen infection [50] (Figure 1). It has recently been reported that in tomato 1-aminocyclopropane-1-carboxylic acid synthase protein (ACS), the rate-limiting enzyme of the ethylene biosynthesis pathway, is regulated by phosphorylation by LeCDPK2 and MAPK after wounding [51]. The phosphorylation/dephosphorylation of LeACS2 regulates its turnover upstream of the ubiquitin-26S-proteasome degradation pathway for the control of ethylene production. Therefore, there seem to be at least two CDPK-signaling pathways acting in a manner that is dependent or independent of phytohormone (JA/ethylene) signaling: the former signaling especially cross-talks with MAPK signaling that strongly contributes to biotic stress-related phytohormone formation [50,52], but the latter does not. Regarding the latter case, it has been proposed recently that in Arabidopsis CDPK and MAPK cascades act differentially in four pathogenesis-mediated regulatory programs to control early genes involved in the synthesis of defense peptides and metabolites, cell wall modifications and redox signaling [53].

## 3. Phytohormone Signaling

The induced plant defenses against herbivores seem to reflect an integrative “cross-talk” between signaling molecules, including Ca^2+^-ions, reactive oxygen species (ROS), protein kinases, JA, *cis*-12-oxophytodienoic acid (OPDA), SA, ethylene, and still unknown members of the octadecanoid family [7,54,55]. In distinct signaling processes, phytohormones such as those noted above play an important role in the transduction of signals. Three phytohormones, SA, JA, and ethylene, are major players in the defense of both monocots and dicots (Figure 1). Genetic and reverse genetic studies have shown that the SA pathway, which plays a major role in both locally expressed basal resistance and systemic acquired resistance (SAR) [56], is primarily activated in response to biotrophic pathogens or insects causing little damage such as phloem-feeding aphids and spider mites [57,58]. In contrast, the JA/ethylene pathway is induced in response to necrotrophic pathogens, wounding, and tissue-damaging insect feeding [55,59].

JA is a signaling molecule, that mediates induced plant responses toward herbivory and pathogen infection, resulting in the activation of distinct sets of defense genes. While JA is known to mediate herbivore resistance, SA mainly mediates pathogen resistance in plants [60]. However, there are some exceptions, for example, plants respond to piercing-sucking herbivores, e.g., aphids, whiteflies and spider mites, by simultaneous up-regulation of SA and JA responses [25,61,62]. mRNAs encoding putative proteins that may be involved in the synthesis of JA and SA are up-regulated in several species of plants infested with aphids, leading to a diversity of plant defense responses, including aphid-dependent blends of plant volatiles (infochemicals), caused by the feeding of various aphid species [63]. Moreover, JA and SA act antagonistically, and are both required for the induced response following herbivore feeding or pathogen attack [64]. JA and SA interact in a mutually antagonistic fashion and JA–SA crosstalk constitutes an excellent example of the complex regulatory networks that allow the plant to fine-tune specific responses to different sets of pathogens [65]. Although several reports suggest overall negative interactions between JA and SA in defense signaling, this cross-talk strongly depends on concentration and timing [66]. Onkokesung *et al.* [67] found an important accessory function of ethylene in the activation of JA-regulated plant defenses against herbivores in *N. attenuata*. JA-ethylene crosstalk restrains local cell expansion and growth after herbivore attack, allowing more resources to be allocated to induced defenses against herbivores [67].

Ethylene is required for the concomitant induction of JA or other signals by modulating the sensitivity to a second signal (*i.e*., Ca^2+^ signal) and its downstream responses [68] (Figure 1). Ethylene seems to play a role as a switch by reducing the production of constitutive defense compounds such as nicotine after herbivore damage and stimulating the production of JA and volatiles [69]. It has also been demonstrated in *Medicago truncatula* that ethylene contributes to the herbivory-induced terpenoid biosynthesis at least twice: by modulating both early signaling events such as cytoplasmic Ca^2+^-influx and the downstream JA-dependent biosynthesis of terpenoids [68].

## 4. JA Signaling via the COI1*-*JAZ Complex

A number of reviews emphasizing different aspects of JA physiology have appeared in recent years focusing on the multifunctional role of the so called “jasmonates” [70]. A combination of genetic, molecular, and biochemical analyses indicates that the core signal transduction chain linking JA synthesis to hormone-induced changes in gene expression consists of a quartet of interacting players: a JA signal, the SCF-type E3 ubiquitin ligase SCF^COI1^, jasmonate ZIM-domain (JAZ) repressor proteins that are targeted by SCF^COI1^ for degradation by the ubiquitin/26*S* proteasome pathway, and transcription factors (e.g., MYC2) that positively regulate the expression of JA-responsive genes [71]. COI1 contains an open pocket that recognizes the JA derivate (3*R*,7*S*)-jasmonoyl-l-isoleucine (JA-Ile, an active form of JA [72]). High-affinity JA-Ile binding requires a bipartite JAZ degron sequence consisting of a conserved α-helix for COI1 docking and a loop region to trap the hormone in its binding pocket [73]. Furthermore, most members of the JAZ gene family in Arabidopsis are highly expressed in response to *Spodoptera exigua* feeding and mechanical wounding [74]. Overexpression of a modified form of JAZ1 (JAZ1Delta3A) that is stable in the presence of JA compromises host resistance to feeding by *S. exigua* larvae [74].

## 5. Involvement of Polyamines

Polyamines are small aliphatic compounds with two or more primary amino group sand are widespread in living organisms. In plants, these compounds have been implicated in a wide range of biological processes including growth and development as well as responses to abiotic and biotic stresses [75–77]. In the case of herbivory stress, it has only been reported that the expression levels of an *S*-adenosylmethionine decarboxylase (SAMDC) gene, involved in polyamine synthesis, is induced in Lima bean leaves in response to attack by spider mites. SAMDC is especially responsible for the synthesis of two polyamines, spermidine and spermine (Spm) [78], but the levels of both of these compounds remain unchanged after herbivory [79]. Exogenous application of Spm to Lima bean leaves induced the emission of volatile organic compounds (VOCs) and stimulated cytoplasmic Ca^2+^ influx. Moreover, simultaneous application of JA and Spm resulted in the release of higher amounts of VOCs than the sum of the separate treatments and the composition of the blend was similar to that induced by spider mites, suggesting synergistic cross-talk between JA and Spm [80].

The production of H_2_O_2_ derived from polyamine oxidation is correlated with cell wall maturation and lignification associated with wound-healing and cell wall reinforcement during pathogen invasion [81,82]. H_2_O_2_ is known to be produced not only from the superoxide anion (O^2 −^) by NADPH oxidase but also through polyamine oxidation by diamine oxidase and polyamine oxidase. The formation of such ROS is one of the earliest plant responses to pathogens, and the ROS trigger downstream reactions. It is postulated that H_2_O_2_ production immediately after the invasion is catalyzed by NADPH oxidases, whereas the later production of H_2_O_2_ results mainly from polyamine oxidation [83]. As described above, ROS, including H_2_O_2_, are also generated massively in the local plant cells in response to herbivory [7,84]. For instance, in *Medicago truncatula* and Lima bean, ROS are generated as a result of herbivory by *Spodoptera litorallis* or spider mites but not by artificial damage [85–87]. H_2_O_2_ may also be generated and function belowground since the expression of a diamine oxidase gene was induced in Arabidopsis roots after inoculation with root herbivore nematodes [88].

Notably, polyamines are frequently conjugated to phenolic compounds and result in formation of phenylpropanoid-polyamine conjugates (PPC). It has been reported that in tobacco plants an R2R3-MYB transcription factor is involved in the regulation of PPC biosynthetic enzymes [89]. Greater mass gain of generalist and specialist herbivorous larvae were found in R2R3-MYB8-silenced tobacco plants compared with their wild-type plants, indicating that activation of PPC biosynthesis is involved in resistance to herbivory by both herbivores [90]. Therefore, it would be interesting to verify whether and how conjugated polyamines, in addition to free polyamines, are involved in resistance to herbivores.

## 6. Airborne Signaling between and within Plants

Along with gaseous phytohormones (e.g., ethylene) induced by herbivory, VOCs including a wide array of low molecular weight terpenes and green leaf volatiles (GLVs) function as airborne signals within and between plants [91,92] (Figure 1). Herbivore-induced VOCs elicit a defensive response in undamaged plants (or parts of plants) under natural conditions, and they function as external signal for within-plant communication, thus also serving a physiological role in the systemic response of a plant to local damage [93]. There is a tendency to interpret plant traits that provide defense against herbivores in terms of their benefits against herbivory. However, those same traits may have many other undescribed consequences [35].

On occasion, receiver plants do not show immediate changes in their level of defenses, but respond stronger and faster than non-receiver plants when damaged by herbivores [94–98]. This readying of a defense response, termed ‘priming’, is demonstrated by the fact that volatiles emitted from clipped sagebrush (*Artimisia tridentata*) affected neighboring *Nicotiana attenuata* plants by accelerating production of trypsin proteinase inhibitors only after *Manduca sexta* larvae started to attack [97]. In hybrid poplar, the expression of genes involved in direct defense was not highly induced in the leaves exposed to one of the GLVs, (*Z*)-3-hexenyl acetate, before herbivory, but was strongly induced once herbivores (gypsy moth larvae) began to feed [95]. Such priming effects were similarly observed in maize plants which had been exposed to VOCs emitted from maize plants infested with generalist herbivores [96]. *Spodoptera littoralis* did not activate genes that are responsive to wounding, JA, or caterpillar regurgitant, but showed primed expression of these genes and reduced caterpillar feeding and development [96].In nature, such volatile-mediated priming may be more significant than volatile-induced resistance following herbivory, because volatile-exposed plants are not certain of the necessity for self-protection against opportunistic pests and may invest in costly defenses only when they are needed [91].The recent finding of rapid methods for selection of mutant plants showing abnormalities in GLVs formation will help to better understand GLVs functional role [99].

The major compounds that are involved in inter/intra-plant communications are two jasmonates (*cis*-jasmone and methyl jasmonate [MeJA] [100,101]), a phenolic compound (methyl salicylate [MeSA] [102]), several terpenes [103,104], and some C_5_–C_10_ alkenals and alkanals, including GLVs: (*E*)-2-hexenal, (*Z*)-3-hexenal, (*Z*)-3-hexenol, and (*Z*)-3-hexenyl acetate[95,105–108]. The history and the nature of emitters and receivers mediated by VOC signals have recently been reviewed [92], but it is difficult to draw conclusions about the common effects of the chemically diverse compounds because previous experiments were performed using several plant species, chemical concentrations, environmental conditions (field or lab), and experimental set-ups.

Some of those volatiles can activate defense genes and this is likely mediated via well-known signaling processes such as Ca^2+^ influx, protein phosphorylation/dephosphorylation and the action of ROS [103,109]. It has been suggested that GLVs that have an α,β-unsaturated carbonyl group can trigger defense through their activity as reactive electrophile species, but other GLVs that have been reported to be biologically active lack this motif [110]. Corn seedlings previously exposed to GLVs or terpenoids from neighboring plants produced significantly more JA and volatile sesquiterpenes when mechanically damaged and induced with caterpillar regurgitate than seedlings not exposed to GLV [111]. Changes in Vm are involved in early signaling events in the cellular response to stress [86,112–114] and exposure to several GLVs changed membrane potentials in intact leaves [115]. It is therefore tempting to speculate that the intra-membrane association of volatiles with membrane proteins, possibly similar to odorant-binding proteins of insects, leads to changes in transmembrane potentials and thereby induces gene activity [116]. However, nothing is known about such sensory proteins for plant volatiles except the gaseous hormone ethylene [91].

Exposure to structurally similar compounds often results in different defensive responses in plants [107,117], suggesting that plants can respond specifically to different chemical compounds or even compounds that differ only in their stereochemistry. The low-molecular-weight, lipophilic nature of numerous VOCs, combined with their vast structural variety and high vapor pressures at ordinary temperatures, account for their role as chemical conveyors of information [118].

## 7. Conclusions

A network of both plant cellular signaling (via phytohormone-dependent/independent pathways) and extra-cellular signaling (via plant VOCs) is induced and modulated in plants in response to herbivory. This highly coordinated and sophisticated network has probably been acquired in order for host plants to respond effectively when damaged by a wide range of feeding attackers. Thus, such complexity appears to exist and act differentially in programs controlling defense genes for acquiring certain kinds of immunity.
